# Developing and evaluating the patient’s perspective of needling questionnaire for haemodialysis

**DOI:** 10.1186/s41687-025-00989-9

**Published:** 2026-01-12

**Authors:** Catherine Fielding, Sarah Brand, Apostolos Fakis, Nicholas M. Selby, Heather Buchanan

**Affiliations:** 1 Research and Innovation and Healthcare of Older People, Nottingham University Hospitals NHS Trust, Nottingham, NG5 1PB England; 2 University of Nottingham, Nottingham, NG7 2RD England; 3 Research, Practice Development & Education and Clinical Research Team Leader, Renal and Transplant Unit, Nottingham University Hospitals NHS Trust, Nottingham, NG5 1PB England; 4 Derby Clinical Trials Support Unit, University Hospitals of Derby and Burton NHS Foundation Trust, Derby, UK; 5 School of Medicine, University of Nottingham, Nottingham, NG7 2RD England; 6 Centre for Kidney Research and Innovation, School of Medicine, University of Nottingham, Nottingham, NG7 2RD England; 7 University Hospitals of Derby and Burton NHS Foundation Trust, Uttoxeter Road, Derby, DE22 3NE England; 8 Lifespan & Population Health, School of Medicine, University of Nottingham, Nottingham, NG5 1PB England

**Keywords:** End stage kidney disease, Haemodialysis, AV access, Cannulation, Patient experience

## Abstract

**Background:**

Cannulation of vascular access for haemodialysis is essential when using arteriovenous access. However, this can be a painful and distressing procedure for patients. There is no current measure that enables inclusion of patients’ experiences of cannulation as an outcome in research comparing interventions to improve cannulation. This study aimed to develop a patient reported outcome measure of cannulation for haemodialysis, focusing on the physical and psychological experiences of patients. This was named the ‘Patient’s Perspective of Needling’ (PPN) questionnaire.

**Methodology:**

The PPN was developed with six patient representatives. Face validity assessed understandability, relevance and comprehensiveness of the PPN. Convergent validity assessed the correlation of the PPN results to the Short-Form Vascular Access Questionnaire (SF-VAQ). Internal consistency and test-retest reliability tested the reliability of the PPN. Floor and ceiling effects were examined and the interpretation of the results of the PPN were explored by: (a) using an item discrimination index; (b) calculating the smallest detectable change. Free text comments were analysed using basic thematic analysis.

**Results:**

The first version of the PPN underwent face validity testing with 12 participants from two renal centres, which led to further amendments of the PPN. The final PPN contained three sections with 17 questions, on pain, worry and problems. Internal consistency of the final PPN was 0.937 (*n* = 98, 95% CI 0.917–0.954, *p* < 0.0001). For convergent validity, a correlation to Question 3 of SF-VAQ was − 0.347 (95% CI -0.146 - -0.521, *p* < 0.001) and Question 4–15 of SF-VAQ was 0.613 (95% CI 0.450–0.736, *p* < 0.001). Test-retest reliability was 0.856 (95% CI 0.788–0.904, *p* < 0.001). The smallest detectable change was 0.135. The Pain section of the PPN scored the highest, with the Worry and Problems sections scoring lower. Free text comments from the PPN expanded on difficulties with cannulation, showing that cannulation experience varied over time and how patients cope with cannulation.

**Conclusions:**

The PPN behaved in a valid and reliable manner for the tests completed, enabling patients’ experiences of cannulation to be included as an outcome in research studies into cannulation for haemodialysis.

**Supplementary Information:**

The online version contains supplementary material available at 10.1186/s41687-025-00989-9.

## Abstract (plain language summary)

Haemodialysis is a treatment for people whose kidneys no longer work. It replaces the function of their kidneys and is essential at least 3 times a week. Some people on haemodialysis require the insertion of large needles into artificially enlarged veins in the arm or leg before each treatment, known as needling, which can be painful and distressing. There is no measure to capture people’s experiences of needling for haemodialysis, meaning this cannot be used as an outcome in research studies aimed to improve needling. We developed the ‘Patients’ Perspective of Needling’ questionnaire to measure physical and psychological consequences of needling for haemodialysis in research studies. This was developed with six patient representatives. We tested this questionnaire in two kidney centres and with a varied sample of people on haemodialysis. Twelve people on haemodialysis assessed the questionnaire for understandability, relevance and comprehensiveness, which led to further changes to the questionnaire. Data were then collected from ninety-eight people on haemodialysis to test other properties of the questionnaire. This was to ensure it measured what it intended to measure (validity), that it did this with consistency (reliability) and to understand what the questionnaire scores meant. The results of these tests showed the questionnaire behaved as it should do and provided guidance on how the questionnaire could be used to capture patients’ experiences of needling for haemodialysis as an outcome in research studies.

**Supplementary Information:**

The online version contains supplementary material available at 10.1186/s41687-025-00989-9.

## Background

Haemodialysis is a life-sustaining treatment for people with end-stage kidney disease. Once initiated, haemodialysis continues for years with four-hour treatments three-times per week, although some patients may need it up to six times a week [1]. Haemodialysis requires special access to the circulation (known as vascular access), which is created surgically. Currently, national and international guidelines recommend that the optimal form of vascular access for the majority of people is an arteriovenous fistula, with an arteriovenous graft the second preference and tunnelled catheter the final option, when other options are not feasible [2–4]. Using an arteriovenous fistula or graft (known as arteriovenous access) requires the insertion of two large needles at the start of each haemodialysis session, known as cannulation, or colloquially as ‘needling’. Cannulation for haemodialysis can be performed by healthcare professionals, carers or by the person themselves.

Cannulation is essential for haemodialysis using arteriovenous access, but this can be a difficult and distressing procedure for some patients. Studies exploring patients’ experiences of cannulation for haemodialysis have found this is a complex experience involving a myriad of physical, psychological and social factors. A recent systematic review found cannulation was associated with pain, concern about abnormal appearance of their vascular access, feeling vulnerable and dependent and worry, about success of cannulation [5]. Other studies have identified that cannulation can be associated with adverse sensations and emotions including pain, anxiety, fear, surrender, loss of control, bodily invasion and altered body image [6, 7]. The cannulator and physical environment can influence patients’ experiences’ of cannulation, as can the individual’s control over the procedure [5–9]. Thus, who performs the cannulation can affect patients’ experiences [7–10]. Studies on the wider experience of haemodialysis [11, 12] and vascular access [9, 13], have again highlighted fear of cannulation. Thus, cannulation can be a distressing but necessary part of haemodialysis.

Despite the distress cannulation can cause patients, there is no current measure to capture patients’ experiences of cannulation for haemodialysis in research. Therefore, patient reported outcomes are rarely included in research studies [14, 15], which risks the symptom burden for patients being missed [16]. A specific, robust and well-validated measure to capture patients’ experiences of cannulation for haemodialysis is needed to enable researchers to measure this as an outcome.

## Aim

This study aimed to develop a patient reported questionnaire of symptoms patients’ experience due to cannulation for haemodialysis, as undertaken by healthcare professionals. The aim was to produce a tool that could be used in research to evaluate interventions focussing on improving cannulation for haemodialysis.

### Methods

We conducted a prospective study to develop a questionnaire that we named the ‘Patient’s Perspective of Needling’ questionnaire (PPN). This focuses on cannulation for haemodialysis, but as experience is affected by who performs the cannulation, this was further defined to focus on cannulation performed by healthcare professionals, rather than self or carer cannulation.

The protocol of the study was registered on the research registry (Researchregistry5243) prior to commencement. The study was approved by Fulham Research Ethics Committee (Reference: 19/LO/1488) and the Health Regulatory Authority (IRAS: 269188). All participants provided informed consent to take part in the study. The study Sponsor was University Hospitals of Derby and Burton NHS Foundation Trust.

### Developing and designing the PPN

The PPN was developed in collaboration with six patient representatives (PRs) from two renal centres, who volunteered to provide their perspective on the design of the questionnaire and conduct of the study. Their involvement was conducted as expected by the Health Research Authority that provides approval for research in the National Health Service in the United Kingdom (UK) (https://www.hra.nhs.uk/planning-and-improving-research/best-practice/public-involvement/). These representatives all had experience of regular cannulation for haemodialysis and varied in gender, ethnicity, age, dialysis vintage and cannulation techniques experienced. PRs were not research participants in the study; their role was purely advisory. All PRs contributed to the content of the questionnaire throughout the study, but sadly two died before data analysis was complete. As this occurred towards the end of the study and in agreement with the remaining PR group, these PRs were not replaced.

The development of the PPN started with a meeting with the PRs and two members of the research team. This was organised to suit PRs, fitting around their haemodialysis schedules. Refreshments were available throughout the day and time and travel expenses were reimbursed, as per INVOLVE guidelines [17]. The content of the day was designed with support from one PR, who provided advice on how to design the meeting and information sharing to promote PR involvement. Prior to the meeting, PRs were given the aims and planned agenda for the day, information on cannulation for haemodialysis, examples of different scales that could be used in the questionnaire (Supplementary Material 1) and a brief description of the findings of a previous systematic review [5]. These were used to structure the discussion surrounding the content of the questionnaire, the scale to be used and the length of recall period for test-retest reliability. PRs were encouraged to drive the meeting and share their thoughts and opinions openly. The meeting provided informal ideas on potential items in the questionnaire, with PRs identifying throughout the meeting what was important to include. Following the meeting, the items to from the questionnaire were drafted by the research team. Themes from the systematic review [5] guided items alongside the PRs discussions from the meeting of what these themes meant to them and what was important to include. Items were reviewed by all PRs in one-to-one meetings, which further refined them and the structure of the questionnaire. Meetings with PRs continued until the content was identified as an adequate representation of relevant points raised by PRs and then finalised to create the PPN version 1 (PPN v1).

The PPN v1 was divided into 4 sections with 22 questions covering ‘pain’, ‘worry’, ‘problems’ and ‘interaction during needling’. Each question could be scored between one and seven. Initially the direction of scoring varied (i.e. for some questions a higher score meant a worse experience and for others a better experience), but in the final PPN all questions scored in the same direction. A scoring strategy was developed, which involved 3 stages to calculate a total PPN score:

1. For those questions where a higher score meant a better experience, these scores were reversed, to ensure all questions scored in the same direction (i.e. 1 = 7, 2 = 6 etc.).
2. The mean of each section was calculated.
3. The means of the sections means was calculated to give an overall PPN score.

This strategy meant:

- The total PPN score was out of seven.
- The total PPN score provided equal emphasis on each section, regardless of the number of questions in it.
- A higher score meant a worse experience.

PRs felt this was the fairest way to represent their experiences.

### Assessment of measurement properties

Assessing measurement properties of a questionnaire can include assessing validity, reliability and responsiveness, as defined by the Consensus-based Standards for the selection of health status Measurement INstruments (COSMIN) reporting standards [18]. Tests of validity assess whether the questionnaire measures the concept it was designed to measure [18–20]. Tests of reliability assess whether the questionnaire measures the concept it was designed to measure consistently. This often equates to stability of the questionnaire, ensuring scores only change when there is change in the construct they are measuring, and not due to error [19]. Responsiveness relates to interpretability of the scores, assessing the ability of the questionnaire to detect change over time and the degree to which meaning can be attributed to scores [18]. This study assesses the measurement properties defined in Table 1.

**Table 1:** Tests completed to assess the measurement properties of the PPN

	Tests Completed	Definitions
Validity	Face Validity	A form of content validity, assessing whether the questionnaire adequately reflects the concept it is designed to measure
	Convergent Validity	Assessing the questionnaire against another questionnaire of a similar concept, to explore if there is expected convergence with the other questionnaire
Reliability	Internal Consistency	Assesses the correlation between answers to individual items of the questionnaire, exploring whether they measure the concept consistently
	Test-Retest Reliability	Assesses whether the questionnaire produces the same results over repeated administrations, when there is no change in the concept being measured. This reflects the stability of the questionnaire over time, ensuring it measures the concept consistently over-time.
Responsiveness and interpretability of scores	Assessment of floor and ceiling effects	Assesses the number of participants who achieved the highest or lowest possible score, identifying redundant items in the questionnaire
	Item discrimination index	Identifies items within the questionnaire that discriminate well between different levels of the concept (e.g. discriminate between good and bad experiences)
	Smallest Detectable Change	The lowest score that can be attributed to a change in the concept and not attributed to error

NB: Content of Table 1 compiled from work by Mokkink et al. [18], Da Souza et al. [19], Polit [20], Cappelleri et al. [21], DeVellis [22], and Terwee et al. [23]

Throughout the research study, questionnaires were given to participants when they were in the haemodialysis unit for their treatment, whilst they were on haemodialysis or waiting to start. Participants were given the option to complete questionnaires either during haemodialysis or take them home and return the completed questionnaire at their next haemodialysis session. When designing the study, PRs felt that dictating when patients completed the questionnaires would add unnecessary burden to participants, who would already be struggling with an intense treatment, haemodialysis. Due to the frequency of cannulation for haemodialysis, PRs also identified that immediacy of the cannulation procedure was unlikely to affect answers, as the maximum time between cannulations was never more than 72 h. Thus, it became more important to minimise burden and promote recruitment of a population inclusive of patients who found haemodialysis burdensome than provide constraints on questionnaire completion.

### Face validity testing of PPN v1

To test face validity, we recruited 12 participants from two renal centres who were undergoing regular cannulation of arteriovenous access for haemodialysis (including haemodiafiltration) performed by healthcare professionals. Participants had to be 18 years old or older and able to complete a questionnaire in English. Purposive sampling was used to gain variation in demographic and clinical characteristics. Participants were asked to complete two questionnaires – the PPN v1 and one to evaluate the content of the PPN (Face Validity Questionnaire, Supplementary Material 2). The Face Validity questionnaire was developed and piloted with PRs before use in the study and produced scores for the understandability, relevance and comprehensiveness of the PPN between one and seven, with a higher score indicating a more positive evaluation. Both questionnaires included free text boxes to enable participants to add comments. The results of face validity testing were reviewed with PRs and further changes were made to create version 2 (PPN v 2).

### Further validity and reliability tests of PPN v2

Once the PPN v2 was finalised, data were collected to assess other measurement properties of the questionnaire. To complete these assessments, we aimed to recruit 80–100 participants across two renal centres, who were undergoing regular cannulation of an arteriovenous fistula or graft for haemodialysis (including haemodiafiltration), performed by healthcare professionals. Whilst sample sizes are rarely defined for validity and reliability tests, Da Souza et al. [19] recommends a sample size of at least 50 for test-retest reliability. To allow for attrition and missing data, a sample size of a maximum of 100 was needed.

Participants were asked to complete three questionnaires in total, across two different time points. They were initially asked to complete the PPN v2 (PPN v2 T1). Alongside this, they were asked to complete the Short-Form Vascular Access Questionnaire (SF-VAQ), which measures vascular access experience [24]. Vascular access experience is a similar concept to cannulation experience but is not the same. The SF-VAQ includes measurement of experiences of types of vascular access that do not undergo regular cannulation prior to haemodialysis and focus on living with the vascular access on a day-to-day basis. Therefore, the impact of specific procedures performed on individual types of vascular access, like cannulation, can be lost. Hence, the comparison of PPN scores to the SF-VAQ was deemed convergent not criterion validity [19, 20, 25]. Convergent validity was assessed against separate sections of the Short-Form Vascular Access Questionnaire (SF-VAQ):

- Question 3 (overall satisfaction with their vascular access), expecting a negative correlation.
- Questions 4–15 (on effect of the vascular access on physical symptoms, social functioning and complications related to their vascular access) expecting a positive correlation.

Participants were asked to complete the PPN a second time (PPN v2 T2), within 14 days of the first PPN completion (PPN v2 T1). This provided data for test-retest reliability, which assess the stability of the questionnaire over time. A short time period was used between PPN v2 T1 and PPN v2 T2, as cannulation for haemodialysis is an unstable procedure that can change over time. Participants were also asked whether the symptoms they had experienced due to cannulation had changed between completion of PPN v2 T1 and PPN v2 T2, using a simple additional question when completing the PPN v2 T2. This question was developed with PRs and provided separately to the PPN v2 T2.

### Statistical analysis

A strategy was developed for missing data. If the answer to more than two questions were missing on any questionnaire, then the participant was excluded from the analysis requiring that questionnaire. If one or two answers were missing, then the mean score of the section was used to replace the question score. To assess this strategy, a secondary analysis of each test was performed, excluding any questionnaires with missing data. A success criterion was also set for missing data, where if more than 20% of data was missing from the PPN questionnaire dataset, this might indicate there was problem, requiring modifications to the PPN.

The clinical and demographic data, the results of the PPN v2 T1 and T2, and SF-VAQ were transcribed and imported into ‘Statistical Package for Social Sciences’ (SPSS) version 24 to enable analysis. Both demographic data and questionnaire answers were found to be non-normally distributed with Shapiro-Wilk test consistently above 0.05. Therefore, medians with inter-quartile ranges were used to describe the distribution of data and non-parametric tests were used throughout the analysis.

The statistical tests used to complete validity and reliability assessments and the required success thresholds are outlined in Table 2. Where appropriate, these tests were analysed for sections of the PPN v2 as well as the total PPN v2, but the analysis of the total PPN v2 was the critical element to determine validity and reliability. A secondary analysis of test-retest reliability was completed only using questionnaires from participants where they felt there was no change in their cannulation. The item discrimination index used the extreme group method which enabled examination of responses to individual items, considering whether they behaved consistently in line with the respondents overall score [21]. Responses to individual items were examined for two groups of participants, the top 25% highest scoring participants (H group) and lowest 25% scoring participants (L group) [21]. This provided a hierarchy of individual questions based on how well they discriminated between those with different experiences. Free text questions were analysed using a basic thematic analysis, following the structure described by Braun and Clarke [26].

**Table 2:** Statistical tests used to assess other measurement properties, with success thresholds

Test	Statistical Method	Success Threshold
Internal Consistency	Cronbach’s Alpha Confidence intervals calculated from intra-class correlation coefficient upper and lower confidence intervals [27]	0.7 or above
Convergent Validity	Spearman’s rho with two-tail significance	0.4–0.7, p-value below 0.05
Test-Retest Reliability	Intra-class correlation coefficient, two-way mixed model effects with absolute agreement [20, 28]	0.5 or above
Floor and Ceiling Effects	Frequency of answers in each category	40% or less in lowest and highest category
Group smallest detectable change for 95% z score	Smallest Detectable Change = Standard Error of Measurement x 1.96 x √2 [29, 30] Standard Error of Measurement = SD Difference/√2 [31]	Less than 1.0

## Results

### Face validity test

The PPN v1 underwent face validity testing with 12 participants across 2 renal centres. Table 3 describes the demographic and clinical characteristics of participants.

**Table 3:** Characteristics of participants completing the face validity test

Site	Site 1	6 (50%)
	Site 2	6 (50%)
Gender	Male	7 (58.3%)
	Female	5 (41.7%)
Age	Median	71 years
	Range	31–90 years
Ethnicity	Caucasian	8 (66.7%)
	Afro-Caribbean	2 (16.7%)
	South Asian	2 (16.7%)
Haemodialysis Vintage (Months)	Median	40 months
	Range	11–128 months
Vascular Access Type	Radiocephalic fistula	4 (33.3%)
	Brachiocephalic fistula	5 (41.7%)
	Brachiobasilic fistula	1 (8.3%)
	Upper arm graft	2 (16.7%)
Canulation Vintage	Median	33 months
	Range	4-128 months
Cannulation Type	Buttonhole	6 (50%)
	Rope Ladder / Area Puncture	6 (50%)
Cause of CKD	Diabetes	4 (33.3%)
	Acute Kidney Injury (AKI)	1 (8.3%)
	Glomerulonephritis	4 (33.3%)
	Vascular Disease	2 (16.7%)
	Unknown	1 (8.3%)
Presence of Diabetes *	Yes	5 (41.7%)
	No	7 (58.3%)
Presence of Vascular Disease **	Yes	7 (58.3%)
	No	5 (41.7%)

* Cause of CKD or Co-Morbidity

**Includes Angina, myocardial Infarction, cerebrovascular disease or peripheral vascular disease

The median score for ease of understanding for all of the four sections of PPN v1 was 7, with all inter-quartile ranges (IQR) between 5 and 7, indicating all sections were easy to understand. The median scores for the relevance of sections varied from 6.5 to 7, with all IQRs between 5.75 and 7, indicating all sections were relevant. The median score for comprehensiveness of the whole PPN v1 was 6 (IQR 6–7), indicating it was comprehensive. The results are summarised in Table 4.

**Table 4:** Results of the face validity questionnaire

Measure	PPN Section	*n*	Median (IQR)
Ease of Understanding	Introduction	12	6.50 (5.25-7.00)
	Pain	12	7.00 (5.25-7.00)
	Worry	11	7.00 (6.00–7.00)
	Problems	11	7.00 (6.00–7.00)
	Interaction during Needling	11	7.00 (6.00–7.00)
Relevance	Pain	11	7.00 (6.00–7.00)
	Worry	10	7.00 (6.00–7.00)
	Problems	10	7.00 (6.00–7.00)
	Interaction during Needling	10	6.50 (5.75-7.00)
Comprehensiveness	Whole PPN	11	6.00 (6.00–7.00)

Free text questions determined whether there were any words or questions participants did not understand. Positive responses included ‘*Very clear*’ (Participant g) and ‘*No words were too difficult*’ (Participant a). However, participants highlighted they did not understand the word ‘*Insertion*’ (Participant f) and individual questions:

*I am given information I need about my needling – not sure what this is asking* (Participant e)

*Interaction – unsure what this section is about* (Participant e).

Overall comments about the PPN v1 included: ‘*Very clear and easy to understand’* (Participant g); ‘*Very quick and easy to complete*’ (Participant e); and ‘*Clear*,* concise and easy to understand*’ (Participant c). Following discussion with PRs, changes were made to the PPN v1 to create the PPN v2. Changes between PPN v1 and v2 included removal of the word ‘insertion’, which was replaced with ‘have your needles put in’ or ‘needling’ where appropriate and the section on the interaction during needling was removed due to ambiguity in how participants interpreted this. In the Further Validity and Reliability tests, it became evident a question around feeling safe during cannulation was difficult to understand, so this was also removed.

### Further validity and reliability tests

The PPN v2 contained three sections with 17 questions, on pain, worry and problems. This became the final version of the PPN (Supplementary Material 3). This phase recruited 99 participants across two renal centres, covering a range of demographic and clinical characteristics summarised in Table 5.

**Table 5:** Characteristics of participants completing questionnaires to assess other measurement properties of the PPN

Site	Site 1	52 (52.5%)
	Site 2	47 (47.5%)
Gender	Male	60 (60.6%)
	Female	39 (39.4%)
Age	Median	69 years
	Range	19–92 years
Ethnicity	Caucasian	81 (81.8%)
	Afro-Caribbean	8 (8.1%)
	South Asian	10 (10.1%)
Haemodialysis Vintage (Months)	Median	31 months
	Range	1-196 months
Canulation Vintage	Median	26 months
	Range	Less than 1–196 months
Cannulation Type	Buttonhole	48 (48.5%)
	Rope Ladder / Area Puncture	50 (50.5%)
	Different each site	1 (1.0%)
Local Anaesthetic Use	Sub-Dermal Lignocaine	5 (5.1%)
	Topical Cream	15 (15.2%)
	Sub-Dermal Lignocaine and Topical Cream	2 (2.0%)
	None	77 (77.8%)
Presence of Diabetes *	Yes	44 (44.4%)
	No	55 (55.6%)
Presence of Vascular Disease **	Yes	26 (26.3%)
	No	73 (73.7%)

* Cause of CKD or Co-Morbidity

**Includes Angina, myocardial Infarction, cerebrovascular disease or peripheral vascular disease

In total, 98 participants completed at least one questionnaire. This provided a study sample of 98 participants, with 96 participants completing the PPN v2 T2 (two participants withdrew due to ill health). In total, 12 PPN v2 T1, 11 PPN v2 T2 and 11 SF-VAQ had missing data and five PPN v2 T1, five PPN v2 T2 and three SF-VAQ were removed completely from the analysis due to missing data. This did not go above the threshold of 20% and there were no trends in the questions with missing data. When analyses were repeated without questionnaires with any missing data, there were minimal changes in results (Supplementary Material 4).

Mild floor effects were present in question 9, 11 and 13 (Table 6), which were questions about ‘worry whether the needles will work well’, ‘worry about the appearance of their fistula or graft’ and ‘worry about bleeding in between dialysis sessions’. However, floor and ceiling effects were absent in the total PPN and section scores.

**Table 6:** Floor and ceiling effects in the PPN

	No. Participants Scoring 1.0-1.99	No. Participants Scoring 6.00–7.00
PPN	18 (18.4%)	3 (3.1%)
Pain	12 (12.2%)	5 (5.1)
Worry	38 (38.8%)	3 (3.1%)
Problems	31 (31.6%)	3 (3.1%)

	No. Participants Scoring 1	No. Participants Scoring 7
Qu 1	8 (8.2%)	8 (8.2%)
Qu 2	4 (4.1%)	21 (21.4%)
Qu 3	30 (30.6%)	2 (2.0%)
Qu 4	7 (7.1%)	15 (15.3%)
Qu 5	36 (36.7%)	3 (3.1%)
Qu 6	35 (35.7%)	18 (18.4%)
Qu 7	32 (32.7%)	9 (9.2%)
Qu 8	31 (31.6%)	11 (11.2%)
Qu 9	46 (46.9%)	6 (6.1%)
Qu 10	32 (32.7%)	10 (10.2%)
Qu 11	45 (45.9%)	7 (7.1%)
Qu 12	32 (32.7%)	8 (8.2%)
Qu 13	51 (52.0%)	2 (2.0%)
Qu 14	38 (38.8%)	14 (14.3%)
Qu 15	24 (24.5%)	5 (5.1%)
Qu 16	33 (33.7%)	2 (2.0%)
Qu 17	21 (21.4%)	3 (3.1%)

Internal consistency, using Cronbach’s Alpha, for the PPN was 0.937 (*n* = 98, 95% CI 0.917–0.954, *p* < 0.0001). Convergent validity was only assessed where there was a PPN v2 T1 questionnaire and SF-VAQ completed on the same occasion. Therefore, 91 participants were included in the convergent validity test. Convergent validity of the PPN to the SF-VAQ showed a correlation to Question 3 of -0.347 (95% CI -0.146 - -0.521, *p* < 0.001) and to Question 4–15 of 0.613 (95% CI 0.450–0.736, *p* < 0.001). Test-retest reliability was assessed in the 88 participants who completed both the PPN v2 T1 and PPN v2 T2. The median time between PPN completions was 4 days (IQR 2–7 days), with the minimum time at 2 days and the maximum time at 14 days. The test-retest reliability for the total PPN was 0.856 (95% CI 0.788–0.904, *p* < 0.001), with both the value and 95% confidence intervals meeting and exceeding the desired threshold. The test-retest reliability slightly increased when removing participants who had a change in their cannulation (*n* = 56, 0.911 (95% CI 0.852–0.946, *p* < 0.0001)). The results of these tests for sections of the PPN is summarised in Supplementary Material 5.

The results of the item discrimination index are shown in Table 7. Questions about overall pain, the frequency of pain, worry about pain, worry about multiple needles attempts, worry about problems with the AV access and worry about who will insert the needles demonstrated good discrimination between different cannulation experiences (Questions 1, 4, 7, 8, 10, 14). No questions demonstrated poor discrimination, and no questions had a negative discrimination index. The standard error of measurement for the PPN was 0.458, providing a group smallest detectable change using a 95% confidence interval of 0.135.

**Table 7:** Item discrimination index

Item	Proportion endorsed from H group	Proportion endorsed from group	Item Discrimination Index
Qu 1	1.0 (100%)	0.04 (4%)	0.96
Qu 7	0.92 (92%)	0	0.92
Qu 8	0.92 (92%)	0.04 (4%)	0.88
Qu 14	0.84 (84%)	0	0.84
Qu 4	0.96 (96%)	0.16 (16%)	0.80
Qu 10	0.80 (80%)	0	0.80
Qu 6	0.76 (76%)	0	0.76
Qu 12	0.76 (76%)	0	0.76
Qu 2	1.0 (100%)	0.36 (36%)	0.64
Qu 5	0.68 (68%)	0.04 (4%)	0.64
Qu 11	0.64 (64%)	0.04 (4%)	0.60
Qu 15	0.68 (68%)	0.08 (8%)	0.60
Qu 3	0.56 (56%)	0	0.56
Qu 17	0.64 (64%)	0.08 (8%)	0.56
Qu 9	0.52 (52%)	0	0.52
Qu 16	0.48 (48%)	0.08 (8%)	0.40
Qu 13	0.28 (28%)	0	0.28

H group is top 25% participants with overall highest PPN score

L group is bottom 25% participants with overall lowest PPN score

An endorsed item was when the participants scored 4–7 in response to that item

### Patients’ experiences of cannulation for haemodialysis

The data from the PPN provided further insight into patients’ experiences of cannulation for haemodialysis. Of the three sections of the PPN, the Pain section scored the highest, indicating this was most problematic for patients, with the Worry and Problems sections scoring lower (Table 8). Questions related to the overall pain from needling, worst pain from needling, frequency of pain and frequency of machine alarms due to the needles scored the highest, with medians of 3 or above. The lowest scoring question was related to worry about bleeding in between haemodialysis sessions.

**Table 8:** Median, minimum and maximum PPN and section scores

	Median	IQR	Minimum	Maximum
Total PPN	3.08	2.07–3.76	1.00	6.56
Pain Section	3.40	2.40–4.80	1.00	7.00
Worry Section	2.39	1.44-4.00	1.00	7.00
Problems Section	2.67	1.67–3.67	1.00	7.00

Participants also provided free text comments related to their experience of cannulation. In total, there were 66 comments from 47 participants, creating three themes and seven sub-themes (Table 9). participants used the free text box to expand on problems with their cannulation (Theme 1). These related to pain, anxiety and problems getting the needles in, coinciding with the three sections of the PPN, expanding or explaining their answers to questions. Many comments indicated that ‘cannulation experience varied’ (Theme 2). Experience could vary with the cannulator, the age of the arteriovenous access and the cannulation technique, with difficulties with cannulation varying between sessions, between participants and also over the longer term. Some comments from participants described how they coped with the cannulation procedure (Theme 3). The whole team of cannulators could have a positive effect on cannulation experience, helping them cope. participants also mentioned how they had become ‘used’ to cannulation, accepting it as an inevitable part of their life.

**Table 9:** Themes and Sub-Themes from free text comments, with related quotes

Theme	Sub-Theme	Quotes
Difficulties with Cannulation	Pain from Cannulation	‘*I do find it all painful and I have to truly convince myself to accept that I have to go for treatment.*’ (Participant au, PPN v2 T1) ‘*Yes it hurts*,* but it is always bearable*’ (Participant y, PPN v2 T2)
	Anxiety about Cannulation	‘*Needling is horrid and makes you anxious beforehand*.’ (Participant g, Face Validity Questionnaire) ‘*The worry about having needles inserted is brief*,* the time it takes to walk into the unit and actually have the needles inserted.*’ (Participant av, PPN v2 T2)
	Problems getting the Needles in	*‘I always have trouble needling my venous. Arterial is OK. I have had lots of blows!’* (Participant an, PPN v2 T1) *'I feel I have a ‘problematic’ graft which can cause issues for people who do not needle me regularly’* (Participant i, PPN v2 T2)
Cannulation experience varies	Cannulation varies with the cannulator	‘*Some people hurt when putting needles in by pressing too hard and re-inserting them. Some people I never feel it at all’* (Participant ab, PPN v2 T1) ‘*Its to who puts needles in. You sometimes hope it is someone who is gentle and some nurses just go for it.*’ (Participant z, PPN v2 T2).
	Cannulation experience varies with the age of the AV access	‘*At the beginning the needles were a problem and machine GOING OFF a lot. After 6 months of being on*,* the staff have done wonders with everything*,* and everything as very much improved.*’ (Participant at, PPN v2 T1)
	Cannulation experience varies with cannulation technique	*‘I liked the buttonhole needling which I could do myself.’* (Participant m, PPN v2 T1) *‘Probably need to visit the removal of scabs which can be painful as much as the needling.’* (Participant aj, PPN v2 T2)
Coping with Cannulation	The cannulation team can inspire trust and improve cannulation	‘*I have complete trust in the team treating me whatever the future’* (Participant z, PPN v2 T1) ‘*All the nurses do a good job and look after us in a brilliant way*’ (Participant x, PPN v2 T2)
	Adapting to Cannulation	*‘Having the needle in is now part of my life that I have gotten used to.’* (Participant ag, PPN v2 T1) *‘Yes it hurts but …. I have to go through what I have to go through so no point in worrying about it*,* just get on with it.*’ (Participant y, PPN v2 T1)

## Discussion

This study developed the Patients’ Perspective of Needling questionnaire (PPN) to measure patients’ experiences of canulation for haemodialysis undertaken by healthcare professionals. This is a patient reported questionnaire that measures symptoms they experience due to cannulation for haemodialysis, as undertaken by healthcare professionals. It is designed to be used within research to measure the impact of interventions on patients’ experiences of cannulation. The PPN was developed with PRs and underwent validity and reliability testing. The results of tests indicate the questionnaire behaves in a valid and reliable manner for the tests completed. The findings indicate that pain is most problematic for patients, with problems with cannulation and worry scoring lower. Free text comments elaborate further on the pain, worry and problems with cannulation they experience; describing how their experience varies with the cannulator, age of the AV access and cannulation technique; and describing how they cope with cannulation.

The PPN is a novel questionnaire that can be used within research to capture patients’ experience of cannulation for haemodialysis, measuring the symptoms patients’ experience due to this procedure. Robust well-validated questionnaires exist to measure patients’ experiences of vascular access for haemodialysis (i.e. Short-Form Vascular Access Questionnaire (SF-VAQ) [24, 32]; Vascular Access Quality of Life questionnaire [33]). However, these questionnaires include types of vascular access that do not undergo regular cannulation prior to haemodialysis and focus on living with the vascular access on a day-to-day basis. Therefore, the impact of specific procedures performed on individual types of vascular access, like cannulation, can be lost. Following completion of PPN development, the Needling Patient Reported Experience Measure (N-PREM) was published [34]. This focusses on measuring patients’ experiences of cannulation to inform clinical practice and quality improvement initiatives, including both self, carer and healthcare professional cannulation with a focus on procedural and psychosocial aspects, rather than the symptoms patients’ experience due to cannulation. The focus of the PPN on symptoms provides a measure that can be used in research to compare interventions, excluding external factors that may influence this beyond the cannulation procedure (e.g. the environment). The PPN remains the first patient reported questionnaire to provide a measure for symptoms patients’ experience due to cannulation for haemodialysis as undertaken by healthcare professionals. Whilst the focus of development of the PPN is for use in research, it also has potential application in day-to-day clinical practice either to improve individuals’ experiences or in quality improvement projects.

This study attempted to recruit a diverse sample that was representative of the haemodialysis population. Whilst only twelve participants took part in the face validity phase, purposive sampling ensured this was diverse, with a wide spectrum of age, vascular access type, haemodialysis vintage and cannulation vintage, and a mix of gender and ethnicity. The sample recruited in the ‘Other Measurement Properties’ phase had similar key characteristics to the normal haemodialysis population, when compared to UK renal registry data [35]. The one exception to this was that both renal centres had a high prevalence of arteriovenous fistula use compared to the UK average. This is unlikely to alter the measurement properties assessed but implies participants may have better than average experience of cannulation, as staff were better practised in this procedure.

Face validity testing of the PPN helped to refine and develop the content of the PPN and ensure this measures what it intends to measure [18–20]. The results of this phase provide reassurance of the understandability, relevance and comprehensiveness of the PPN, essential parts of ensuring content validity [25]. Moreover, other elements of the study also provide reassurance of the validity of the PPN. Content validity is also affected by how the questionnaire is developed [22]. Involving PRs throughout the development of the PPN ensured the content of the PPN remained grounded in patients’ true experiences. The results of convergent validity tests against the SF-VAQ provide further reassure of the PPN’s validity. Specific issues with validity of certain questions were identified throughout testing of the PPN, related to complex concepts that were interpreted in different ways by different people (i.e. the interaction during needling and feeling safe). To ensure validity, these questions were removed from the final PPN, though these concepts remain valid parts of patients’ experiences of cannulation [5, 36, 37]. Therefore, it is recommended that studies into cannulation should consider a mixed methods approach where more complex concepts such as safety and interaction with the cannulator can be explored in semi-structured interviews.

The reliability of the PPN, which tests whether the questionnaire measures what it is designed to measure consistently [19], was assessed using internal consistency, using Cronbach’s Alpha, and test-retest reliability tests. The results of these tests provided reassurance of the PPN’s reliability and excelled beyond success thresholds. However, it has been raised that internal consistency using Cronbach’s Alpha above 0.9 or 0.95 can demonstrate a problem with the questionnaire, where there is too much homogeneity indicating redundant items [38]. This was considered, but no repetition was identified across items. PRs continued to report all elements were relevant and different. Using absolute agreement for intra-class correlation requires a strict agreement between the two questionnaire completions [22], providing further reassurance of reliability. It should be recognised, however, that the time between questionnaire completions was short, increasing the chance participants remembered their answers between the two completions. This demonstrates the caution with which reliability results should be interpreted, where no single test is viewed in isolation, but each test contributes to the whole picture of validity and reliability.

The completed tests on the PPN of measurement properties also facilitate interpretation of the PPN scores and indicate how they should be used within research. Firstly, the overall PPN demonstrated better validity and reliability than section scores. Therefore, whilst section scores may be useful to add granularity and understanding to data, the overall PPN score is the score of most interest and relevance to research. Secondly, the items discrimination index demonstrated those questions that best discriminated between those with a negative and less negative experience. Whilst the whole questionnaire is the optimal way to determine whether interventions make a difference to patients’ experiences of cannulation, answers to individual questions may provide indication of how the interventions improve their experience and add granularity to data. Thirdly, the PPN had a narrow ‘Group smallest detectable change’, indicating that a change in score over 0.14 when comparing research groups may indicate a clinically meaningful change. However, the smallest detectable change is not the same as a clinically meaningful change but does set a threshold that the clinically meaningful change must be above [25]. Further work needs to be done to identify what is a clinically meaningful change.

The results of the PPN within this study also provide novel insight into patients’ experiences of cannulation for haemodialysis. Unsurprisingly, the Pain section scored highest, but the Worry and Problems with cannulation sections provided new measures for these concepts. All the sections of the PPN have congruence with previous qualitative research [5, 6, 39]. The low score of the question about bleeding in between dialysis is likely to be a reflection of the rarity of this event. PRs felt this remained relevant to include as when it happened, it was traumatic for patients. However, the variability in cannulation experience described in free text comments was a novel finding. These comments indicated that for individuals’ experiences varied over time and between haemodialysis sessions, suggesting that different and varied factors can affect patients’ experiences of cannulation, including the cannulator, the quality of the access and the cannulation technique. Whilst individual studies have identified variation in different cannulators [40] and the difference cannulation technique can make to patients’ experiences [41], no research has examined variation in cannulation or what causes this in-depth. Further research is needed to understand this variability.

### Limitations

For this study we did not complete assessment of all the measurement properties described by COSMIN [18] and made a pragmatic decision the assessments completed based on the time and resources available. A factor analysis to confirm structural validity was considered but not performed. Initially an exploratory factor analysis was not considered worthwhile as it was expected the questionnaire would have 5–10 question covering one simple concept. However, the final PPN was more complex than initially envisioned. At this stage the sample size was not large enough to perform a confirmatory factor analysis, although it is unclear whether this would add to easily defined sections. The PPN was also only assessed in English and future studies are needed to test cross-cultural validity. We also did not assess divergent validity or assess stability using an external measure. Finally, no longitudinal data were available to assess responsiveness over time, which requires exploration given the discovery of variation in patients’ experiences of cannulation over time. Further research may be required to assess these elements and add to the rigor to the PPN development. Therefore, the PPN has demonstrated validity and reliability, but only within context of the tests completed.

There were also limitations to the way tests were conducted. We chose face validity to ensure the patient’s interpretation of the questionnaire was the main focus. However, exploring content validity with a broader group of experts may have provided greater reassurance [18]. Using a questionnaire to assess face validity lacks depth, and the use of cognitive interviewing may have provided more depth and reassurance of validity [42]. Items in the PPN were determined by discussion with PRs, but this was not done using research methodology. Therefore, PRs opinions may have been influenced by the research team. Whilst we attempted to dissuade agreement with our perspectives unless this was true, we cannot exclude this influence.

## Conclusions

The study has developed and assessed the measurement properties of the Patients’ Perspective of Needling (PPN) questionnaire for cannulation for haemodialysis. The PPN behaved in a valid and reliable manner for the test completed. The PPN is a patient reported questionnaire that measures symptoms patients’ experience due to cannulation for haemodialysis, as undertaken by healthcare professionals, enabling patients’ experiences of cannulation to be included as an outcome in research studies into cannulation for haemodialysis. Administering the PPN in the context of a mixed methods study will allow more complex elements of patients’ experiences of cannulation to also be explored. Whilst the PPN enabled measurement of known concepts like pain, worry and problems with cannulation, free texts comments identified that variation happens in cannulation for individuals, over time and between haemodialysis sessions. This novel finding requires further investigation to understand it fully.

## Supplementary Information

Below is the link to the electronic supplementary material.

Supplementary Material 1

Supplementary Material 2

Supplementary Material 3

Supplementary Material 4

Supplementary Material 5

## Data Availability

The datasets generated and/or analysed during the current study are not publicly available due to not all participants consenting for their individual data to be shared with other researchers for further research studies.

## Abbreviations

COSMIN: Consensus-based Standards for the selection of health status Measurement INstruments
N: PREM-Needling Patient Reported Experience Measure
PPN: Patient’s Perspective of Needling questionnaire
PPN v1: PPN version 1
PPN v2: PPN version 2
PPN v2 T1: PPN version 2 at time point 1
PPN v2 T2: PPN version 2 at time point 2
PR: Patient Representative
PREM: Patient Reported Experience Measure
PROM: Patient Reported Experience Measure
SF: VAQ-Short Form Vascular Access Questionnaire
