# Supplementary material for: Developing and evaluating the patient’s perspective of needling questionnaire for haemodialysis
Source: J Patient Rep Outcomes. 2026 Jan 12;10:19. doi: 10.1186/s41687-025-00989-9 (PMC12886701; doi:10.1186/s41687-025-00989-9)
Supplement: Supplementary file 1 — Supplementary Material 1 [file 41687_2025_989_MOESM1_ESM.pdf]

A Study to Develop a Questionnaire to Capture Patients' Perspectives of Cannulation of Arteriovenous Access for Haemodialysis

HD Cannulation PRO – Patient Representative Meeting

Date: XXX **Time:** 11.30am-4.30pm

Location: XXX

Attendees: XXX

Agenda:

1) Introductions
2) Aims of the day
3) What is important to include
4) What scale to use
5) What needs to be included in the introduction
6) Payment details

Breaks:

Lunch 1-1.45pm

Tea Break 3-3.30pm

Aims of Today

- The study aims to develop a questionnaire to capture patients' views of needling
  - Specific to haemodialysis

- The questionnaire is for the purposes of research
  - Be able to define between different techniques
  - About assessing techniques, not individual staff

- The questionnaire will capture people's views which can also be described as their experience or their perspective. This can include:
  - Symptoms during the needling e.g. pain
  - How they felt before, during and after the needling e.g. feel worried
  - Whether the patient feels the needling is successful e.g. works well for haemodialysis
  - How the needling makes their fistula or graft look
  - Whether the needling causes them more problems / complications
  - It is about how the needling technique impacts them as a haemodialysis patient

- The questionnaire will be written by XXX, after today's meeting
  - I will use your thoughts and opinions to shape this and will try to remain true to these
  - It may not end up exactly as you wish
  - I will ask you to look at this, to get your thoughts
  - There may be aspects I cannot change, however much you want them to change, due to the needs of research
  - Please do not let any of this stop you being honest

- Once XXX is happy with the questionnaire, it will undergo a number of tests to make sure it is correct
  - These tests make sure it is good enough to use in research and that people will accept the answers
  - These tests make sure the questionnaire works for different patients in different situations
  - This may lead to further changes

Buttonhole Needling involves inserting the needle at each site in exactly the same manner each time. The needle enters the same hole in the skin and vein, in exactly the same direction and depth each time. Normally buttonhole needling involves using the same needling site each time. In some cases, patients may have 3 or 4 needling sites, so that sites can be rested, especially if patients dialyse 4-6 times a week.

Prior to inserting the needle, the scab from the previous needling site needs to be removed. Once buttonhole needling has been used for a few sessions, needling is changed to use 'blunt' / 'dull' needles.

A fistula that has undergone buttonhole needling will only have 2-4 puncture sites on the arm and often looks like this:

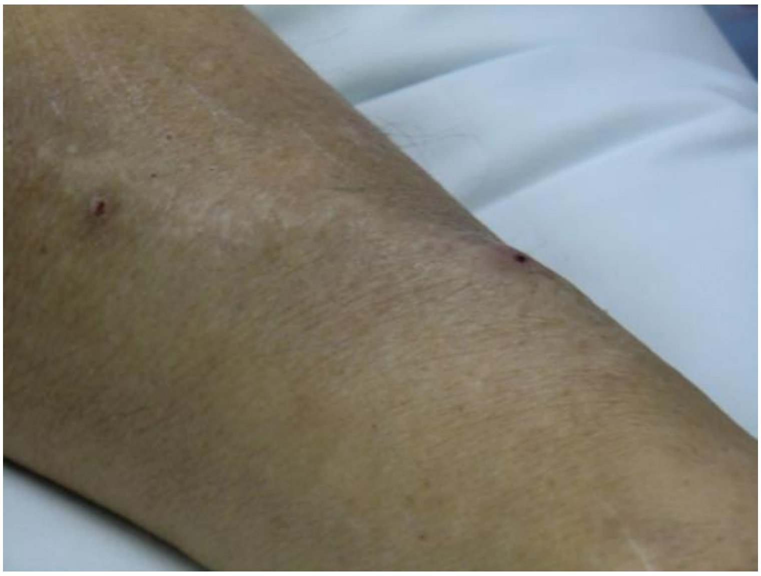

Current advantages include:

- Less long-term damage to the fistula
- Easier needle insertion
- Less bruising from needle insertion
- Better appearance of the fistula with no bulges
- Encourages self-needling

Current problems mainly focus on infection. Some research claims buttonhole needling is more painful and leads to more problems with needle insertion.

<u>Rope Ladder Needling</u> involves needling in a different site each time. However, each needling site is planned so that each needling happens just above the previous needling site. The planned needling sites look like:

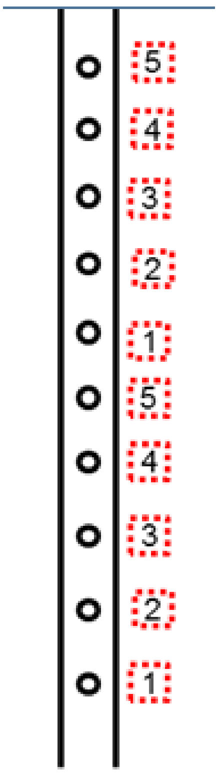

The first needling starts at site 1, then the next needling happens at site 2 and so on.

Once the top of the vein is reached, needling starts again at the bottom of the vein.

Current advantages are:

- Less long-term damage to the fistula
- Lowest infection risk.

Current problems include:

- Bulges developing on the fistula
- Problems inserting the needles
- Bruising on the fistula from needling.

Rope ladder is far less well researched than buttonhole needling.

Likert Scales

- Likert scales have 5 or 7 points what people need to determine where they sit
- They can also have 4 points and remove the ‘Neutral’ point
- The CKD PREM uses Likert Scales

Scales can be:

	0	1	2	3	4	5	6
Agree - Disagree	Strongly disagree	Moderately disagree	Slightly disagree	Neutral	Slightly agree	Moderately agree	Strongly agree
Satisfied - dissatisfied	Completely dissatisfied						Completely satisfied
Never – Always	Never						Always
Negative - Positive	Negative						Positive
Very bad – Very good	Very bad						Very good
Important - Unimportant	Not important at all						Very Important
Excellent - Poor	Poor						Excellent

Almost always true – Almost never true	Almost never true						Almost always true
Definitely – Definitely Not	Definitely Not						Definitely
Helpful - Not Helpful	Not Helpful						Helpful
Safe - Unsafe	Unsafe						Safe

Rating Scales

0 ____ 100

0 ____ 10

I___I___I___I___I___I___I___I___I___I___I

0 10 20 30 40 50 60 70 80 90 100

I___I___I___I___I___I___I___I___I___I___I

0 1 2 3 4 5 6 7 8 9 10
