# Supplementary material for: Developing and evaluating the patient’s perspective of needling questionnaire for haemodialysis
Source: J Patient Rep Outcomes. 2026 Jan 12;10:19. doi: 10.1186/s41687-025-00989-9 (PMC12886701; doi:10.1186/s41687-025-00989-9)
Supplement: Supplementary file 2 — Supplementary Material 2 [file 41687_2025_989_MOESM2_ESM.docx]

**P**atients’ **P**erspectives of **N**eedling for Haemodialysis

Your Opinions on the PPN Questionnaire

We have asked you to complete the PPN questionnaire. Now we would like to gain your thoughts about this questionnaire. We would like to know how easy this was to understand and how well it reflects your opinions and views on your needling.

We have spilt the PPN questionnaire into different sections. The different sections are:

1. Pain – questions 1-5
2. Worry – questions 6 – 13
3. Problems – questions 14-17
4. Interaction during needling – questions 18-22

Some of the questions will be asking about the different sections in the PPN questionnaire. If you do not understand the different sections in the PPN questionnaire, please ask the research team to explain this.

Where a scale is used for questions, please rate each of your answers between 1 and 7, by marking an’ X’ on the scales. Your answers to these questions will be anonymous. Please answer honestly – your opinion is valuable to us and it is important that we know if the questionnaire is wrong in anyway or if it is right. Please answer all questions, even if you believe the PPN questionnaire is correct.

You will see the questionnaire starts with some general questions. There will be the opportunity to provide more detail in later questions.

Please ensure you have completed the PPN questionnaire before completing this one. You may want to refer to the PPN questionnaire when completing this one.

Please do not complete the participant ID number below – the research team will do this.

**Participant ID No.** _________

First of all, we would like to know how easy the questions in the PPN questionnaire were to understand.

Please indicate below, by putting a ‘X’ in the box, how easy the questions **in each of the sections** were to understand
(1= Not easy to understand at all; 7=I could understand everything)

|  | Did not understand at all | | | | Easy to understand | | |
| --- | --- | --- | --- | --- | --- | --- | --- |
|  | 1 | 2 | 3 | 4 | 5 | 6 | 7 |
| 1. Pain Section (Qu 1-5) |  |  |  |  |  |  |  |
| 1. Worry Section (Qu 6-13) |  |  |  |  |  |  |  |
| 1. Problems Section  (Qu 14-17) |  |  |  |  |  |  |  |
| 1. ‘Interaction during needling’ Section (Qu 18-22) |  |  |  |  |  |  |  |

1. How easy were the introduction and instructions (first page) to understand?

| Not easy at all | | | | Very Easy | | |
| --- | --- | --- | --- | --- | --- | --- |
| 1 | 2 | 3 | 4 | 5 | 6 | 7 |
|  |  |  |  |  |  |  |

| 1. Were there any **words** in the introduction and instructions you did not understand?   Please write any words you did not understand below. |
| --- |
|  |

For the questions below, please write the question number you **did not** understand. Please feel free to add any comments on this in the box.

| 1. Were there any questions in the pain section (Qu 1-5) you did not understand or that did not make sense?   If so, please write which questions. |
| --- |
|  |
| 1. Were there any questions in the worry section (Qu 6-13) you did not understand or that did not make sense?  If so, please write which questions. |
|  |
| 1. Were there any questions in the problems section (Qu 14-17) you did not understand or that did not make sense?  If so, please write which questions. |
|  |
| 1. Were there any questions in the ‘Interaction during needling’ Section (Qu 18-22) you did not understand or that did not make sense?  If so, please write which questions. |
|  |

For the questions below, please write the **words** you did not understand. Please feel free to add any comments on this in the box.

| 1. Were there any words in the pain section (Qu 1-5) you did not understand?  If so, please write which words. |
| --- |
|  |
| 1. Were there any words in the worry section (Qu 6-13) you did not understand? If so, please write which words. |
|  |
| 1. Were there any words in the problems section (Qu 14-17) you did not understand? If so, please write which words. |
|  |
| 1. Were there any words in the ‘Interaction during needling’ section  (Qu 18-22) you did not understand?  If so, please write which words. |
|  |

Secondly, we would like to know whether the PPN questionnaire properly captures your views and opinions on your needling for haemodialysis.

Please indicate below how **relevant** the questions in each of the sections were to your needling experience:

|  | Not relevant at all | | | | Completely relevant | | |
| --- | --- | --- | --- | --- | --- | --- | --- |
|  | 1 | 2 | 3 | 4 | 5 | 6 | 7 |
| 1. Pain Section (Qu 1-5) |  |  |  |  |  |  |  |
| 1. Worry Section (Qu 6-13) |  |  |  |  |  |  |  |
| 1. Problems Section  (Qu 14-17) |  |  |  |  |  |  |  |
| 1. ‘Interaction during needling’ Section (Qu 18-22) |  |  |  |  |  |  |  |

1. How much did the questionnaire completely reflect all of your views and opinions on your needling for haemodialysis:

| Not at all | | | | Completely | | |
| --- | --- | --- | --- | --- | --- | --- |
| 1 | 2 | 3 | 4 | 5 | 6 | 7 |
|  |  |  |  |  |  |  |

For the questions below, please write the question number you felt was **not relevant**. Please feel free to add any comments on this in the box.

| 1. Were there any questions in the pain section (Qu 1-5) that were **not relevant** to your needling?  If so, please write which questions. |
| --- |
|  |
| 1. Were there any questions in the worry section (Qu 6-13) that were **not relevant** to your needling?   If so, please write which questions. |
|  |
| 1. Were there any questions in the problems section (Qu 14-17) that were **not relevant** to your needling? If so, please write which questions. |
|  |
| 1. Were there any questions in the ‘Interaction during needling’ Section  (Qu 18-22) that were **not relevant** to your needling?  If so, please write which questions. |
|  |

For the questions below, please write below anything you feel was **missing** from each of the sections in the PPN questionnaire.

| 1. Were there any aspects of your views and opinions on your needling missing from the pain section (Qu 1-5)? |
| --- |
|  |
| 1. Were there any aspects of your views and opinions on your needling missing from the worry section (Qu 6-13)? |
|  |
| 1. Were there any aspects of your views and opinions on your needling missing from the problems section (Qu 14-17)? |
|  |
| 1. Were there any aspects of your views and opinions on your needling missing from the ‘Interaction during needling’ Section (Qu 18-22)? |
|  |

1. Was there anything else you felt was missing from the PPN questionnaire or you would recommend adding?

If you would like to comments on the PPN questionnaire, please write in the box below.

Thank you for taking the time to complete this questionnaire.
