# Supplementary material for: Developing and evaluating the patient’s perspective of needling questionnaire for haemodialysis
Source: J Patient Rep Outcomes. 2026 Jan 12;10:19. doi: 10.1186/s41687-025-00989-9 (PMC12886701; doi:10.1186/s41687-025-00989-9)
Supplement: Supplementary file 3 — Supplementary Material 3 [file 41687_2025_989_MOESM3_ESM.pdf]

Date ___/___/_____

Patients' **P**erspectives of **N**eedling for Haemodialysis

PPN Questionnaire

This questionnaire is designed to capture your views and opinions on your needling before haemodialysis. We recognise that this can vary and is complicated, so please answer questions as best you can.

When completing this, please think about how it was to have your needles put in over the **last 4 weeks**, rather than just what has happened today.

The questionnaire is divided into 3 sections. Please rate each of your answers between 1 and 7, by marking an' X' on the scales. We hope this questionnaire will only take you 5-10 minutes to complete. There is box at the end of the questionnaire where you can add further any comments, if you would like to.

Your answers to these questions will be anonymous. Please answer honestly.

Please do not complete the participant ID number below – the research team will do this.

Participant ID No. _________

© University Hospitals of Derby and Burton NHS Foundation Trust 2025. All rights reserved. Not to be reproduced in whole or in part without the permission of the copyright owner.
Fielding, Brand, Fakis, Selby and Buchanan

Pain

We understand that having the needles put in before haemodialysis can be painful. We would like to understand a little bit more about this. In particular we would like to know how bad this is for you, how it might vary and how often you have felt pain, in the last 4 weeks.

1) Most of the time, how **painful** is it to have your needles put in?

No Pain at all						Very Painful
1	2	3	4	5	6	7

2) Think about your **worst** experience of needling in the last 4 weeks. How painful was this?

No Pain at all						Very Painful
1	2	3	4	5	6	7

3) Think about your **best** experience of needling in the last 4 weeks. How painful was this?

No Pain at all						Very Painful
1	2	3	4	5	6	7

4) How **often** is it painful to have the needles put in?

Never						Always
1	2	3	4	5	6	7

5) How much pain do you normally get from your needles **during** your haemodialysis?
(This pain may start whilst the needles are being put in and remain, or it may start later in your treatment).

No Pain at all						Very Painful
1	2	3	4	5	6	7

© University Hospitals of Derby and Burton NHS Foundation Trust 2025. All rights reserved. Not to be reproduced in whole or in part without the permission of the copyright owner.
Fielding, Brand, Fakis, Selby and Buchanan

Worry

We understand that haemodialysis patients can be worried about having their needles put in before haemodialysis. We would like to understand how much you have worried about your needling and what you worry about, in the last 4 weeks.

6) How **often** do you worry about having your needles put in?

Never						Always
1	2	3	4	5	6	7

Please indicate below how **much** you worry or are concerned about the following things:

	No Worry at all						Very Worried
	1	2	3	4	5	6	7
7) I worry whether the needling will be painful							
8) I worry whether they will have more than one try to put my needle in							
9) I worry whether the needles will work well for my treatment							
10) I worry whether I will get problems with my fistula / graft							
11) I worry about the appearance of my fistula / graft							
12) I worry about bleeding from my fistula / graft at the end of haemodialysis							
13) I worry about bleeding in between haemodialysis sessions							
14) I worry about who is going to put my needles in							

© University Hospitals of Derby and Burton NHS Foundation Trust 2025. All rights reserved. Not to be reproduced in whole or in part without the permission of the copyright owner.
Fielding, Brand, Fakis, Selby and Buchanan

Problems

We know that you can experience problems from your needles. We would like to understand and how often you feel problems have happened in the last 4 weeks. We would also like to know whether you feel safe when the needles are put in.

Please indicate below, how **often** you feel the following problems happen:

	Never						Always
	1	2	3	4	5	6	7
15) Try more than once to get the needle in							
16) Bruising from having the needles put in							
17) The machine alarms in treatment due to the needles							

We recognise this questionnaire may not reflect all of your views and opinions on your needling. If you would like to comment anything, please write in the box below.

Thank you for taking the time to complete this questionnaire.

© University Hospitals of Derby and Burton NHS Foundation Trust 2025. All rights reserved. Not to be reproduced in whole or in part without the permission of the copyright owner.
Fielding, Brand, Fakis, Selby and Buchanan
