# Supplementary material for: Developing and evaluating the patient’s perspective of needling questionnaire for haemodialysis
Source: J Patient Rep Outcomes. 2026 Jan 12;10:19. doi: 10.1186/s41687-025-00989-9 (PMC12886701; doi:10.1186/s41687-025-00989-9)
Supplement: Supplementary file 4 — Supplementary Material 4 [file 41687_2025_989_MOESM4_ESM.docx]

Supplementary Material 2 – Effect of replacing Missing Data on Results

|  | PPN with Missing Data Replaced (1-2 qus only) | | PPN with no missing data | |
| --- | --- | --- | --- | --- |
|  | n | Median (IQR) | n | Median (IQR) |
| **PPN T1** | | | | |
| Total PPN | 93 | 3.06 (2.09-3.76) | 86 | 2.99 (2.07-3.76) |
| Pain Section | 97 | 3.40 (2.40-4.80) | 86 | 3.60 (2.40-4.80) |
| Worry Section | 97 | 2.44 (1.44-4.00) | 86 | 2.28 (1.41-4.00) |
| Problems Section | 97 | 2.67 (1.67-3.67) | 86 | 2.83 (1.67-3.67) |
| **PPN T2** | | | | |
| Total PPN | 93 | 2.88 (1.98-3.96) | 85 | 2.87 (1.99-3.95) |
| Pain Section | 95 | 3.20 (2.40-4.60) | 85 | 3.20 (2.40-4.60) |
| Worry Section | 95 | 2.56 (1.44-4.06) | 85 | 2.56 (1.44-4.00) |
| Problems Section | 95 | 2.33 (1.67-6.67) | 85 | 2.33 (1.67-3.67) |

|  | PPN with Missing Data Replaced (1-2 qus only) | | PPN with no missing data | |
| --- | --- | --- | --- | --- |
|  | n | Test Result | n | Test Result |
| Internal Consistency | 98 | 0.937 (0.917-0.954) (p<0.001) | 86 | 0.937 (0.915-0.955) (p<0.001) |
| Convergent Validity with SF-VAQ Qu 3 | 91 | 0.347 (0.146-0.521) (p=0.001) | 84 | 0.343 (0.132-0.524) (p=0.001) |
| Convergent Validity with SF- VAQ Qu 4-15 | 90 | 0.613 (0.450-0.736) (p<0.001) | 80 | 0.635 (0.478-0.753) (p<0.001) |
| Test-Retest Reliability | 88 | 0.856 (0.788-0.904) (p<0.001) | 73 | 0.838 (0.751-0.896) p<0.001) |
