# Supplementary material for: Developing and evaluating the patient’s perspective of needling questionnaire for haemodialysis
Source: J Patient Rep Outcomes. 2026 Jan 12;10:19. doi: 10.1186/s41687-025-00989-9 (PMC12886701; doi:10.1186/s41687-025-00989-9)
Supplement: Supplementary file 5 — Supplementary Material 5 [file 41687_2025_989_MOESM5_ESM.docx]

Supplementary Material 3 – Analyses of Sections of PPN

**Internal Consistency**

|  | Number of Qus | Desired Threshold | Cronbach Alpha | 95% CI | p value |
| --- | --- | --- | --- | --- | --- |
| Total PPN | 17 | Above 0.7 | 0.937 | 0.917 - 0.954 | <0.001 |
| Pain Section | 5 |  | 0.877 | 0.833 - 0.911 | <0.001 |
| Worry Section | 9 |  | 0.932 | 0.910 – 0.950 | <0.001 |
| Problems Section | 3 |  | 0.748 | 0.648 – 0.824 | <0.001 |

*Internal Consistency results for total PPN and sections*

**Convergent Validity**

|  | Desired Threshold | r | 95% CI | p value |
| --- | --- | --- | --- | --- |
| Total PPN to SF-VAQ Qu 3 | 0.4-0.7 | 0.347 | -0.146-0.521 | 0.001 |
| Total PPN to SF-VAQ Qu 4-15 |  | 0.613 | 0.450-0.736 | <0.001 |
| Pain Section to SF-VAQ Qu 4-15 |  | 0.494 | 0.308-0.643 | <0.001 |
| Worry Section to SF-VAQ Qu 4-15 |  | 0.617 | 0.455-0.740 | <0.001 |
| Problems Section to SF-VAQ Qu 4-15 |  | 0.411 | 0.215-0.575 | <0.001 |

*Convergent Validity between SF-VAQ sections and PPN and sections*

**Test-Retest Reliability**

|  | Desired Threshold | Including all Participants | | Only including participants with no change in their cannulation | |
| --- | --- | --- | --- | --- | --- |
|  |  | n | Intra-class correlation | n | Intra-class correlation |
| Total PPN | Above 0.5 | 88 | 0.856 (0.788-0.904) (p<0.001) | 56 | 0.911 (0.852-0.946) (p<0.001) |
| Pain Section |  | 88 | 0.854 (0.772-0.906) (p<0.001) | 56 | 0.846 (0.743-0.909) (p<0.001) |
| Worry Section |  | 88 | 0.806 (0.718-0.869) (p<0.001) | 56 | 0.867 (0.784-0.920) (p<0.001) |
| Problems Section |  | 88 | 0.688 (0.560-0.783) (p<0.001) | 56 | 0.766 (0.631-0.856) (p<0.001) |

*Test-Retest Reliability of PPN and sections, with analysis only including participants with no perceived change in their cannulation between PPN T1 and PPN T2*
